# Experimental evolution under colistin selection increases colistin minimum inhibitory concentration (MIC) without detectable collateral MIC shifts in *Escherichia coli* ATCC 25922

**DOI:** 10.1038/s41598-026-56362-3

**Published:** 2026-06-05

**Authors:** Ádám Kerek, Bence Török, Levente Laczkó, Gábor Kardos, Krisztián Bányai, Eszter Kaszab, Krisztina Bali, Ákos Jerzsele

**Affiliations:** 1 Department of Pharmacology and Toxicology, University of Veterinary Medicine Budapest, Budapest, 1078 Hungary; 2 National Laboratory of Infectious Animal Diseases, Antimicrobial Resistance, Veterinary Public Health and Food Chain Safety, University of Veterinary Medicine Budapest, Budapest, 1078 Hungary; 3 One Health Institute, University of Debrecen, Nagyerdei krt. 98, Debrecen, 4032 Hungary; 4 HUN-REN–UD Conservation Biology Research Group, Egyetem tér 1, Debrecen, 4032 Hungary; 5 National Public Health Center, Albert Flórián út 2-6, Budapest, 1097 Hungary; 6 Department of Gerontology, Faculty of Health Sciences, University of Debrecen, Sóstói út 2-4, Nyíregyháza, 4400 Hungary; 7 Department of Medical Biology, Medical School, University of Pécs, Pécs, 7624 Hungary; 8 HUN-REN Veterinary Medical Research Institute, Budapest, 1143 Hungary; 9 Department of Microbiology and Infectious Diseases, University of Veterinary Medicine, István u 2, Budapest, 1078 Hungary

**Keywords:** Experimental evolution, Colistin-specific adaptation, Polymyxins, *Escherichia coli*, Antimicrobial resistance, Spatial selection gradient, Genetics, Microbiology

## Abstract

Colistin is a last-resort antimicrobial in human and veterinary medicine, yet it remains unclear whether prolonged exposure can drive collateral resistance beyond polymyxins. Here, we used a spatial experimental-evolution platform, the Microbial Evolution and Growth Arena (MEGA)-plate, to examine adaptation of *Escherichia coli* ATCC 25922 to stepwise colistin selection in three independent runs. Endpoint isolates from all three runs (*n* = 3 isolates per exposure zone) were phenotyped, whereas one randomly selected representative isolate per exposure zone (0× , 1× , 10× , 100× , and 1000×) was sequenced because MIC profiles were identical across runs. Colistin MIC increased from 0.5 to 64 μg/mL across the selection gradient, while MICs for the other tested agents remained unchanged. Across the sequenced representatives, no plasmid-mediated colistin resistance determinants were detected and the overall set of CARD strict hits remained unchanged. Comparative genomics identified only limited coding changes with potential relevance to envelope-associated adaptation, notably an *ftsI* missense variant in the 100× and 1000× representatives and an *ompC* synonymous variant of uncertain functional significance in the 1× representative. Relative to the untreated representative, exposed isolates differed by only a small number of baseline-unique high-confidence annotated coding variants. These data support a compound-restricted phenotypic response in this single-strain model under the conditions tested. However, because genomic sampling was limited to one endpoint representative per exposure zone and no transcriptomic or functional assays were performed, broader regulatory adaptation or efflux-related responses cannot be excluded.

**Supplementary Information:**

The online version contains supplementary material available at 10.1038/s41598-026-56362-3.

## Introduction

Polymyxins are cationic cyclic lipopeptides produced by *Paenibacillus polymyxa* and comprise five chemically related compounds (A–E), of which polymyxin B and colistin (polymyxin E) are currently used in clinical practice^1^. Polymyxins exert their antibacterial activity primarily through disruption of the outer membrane of Gram-negative bacteria, leading to membrane destabilization and cell death^2^.

The primary target of polymyxins is lipopolysaccharide (LPS), a key structural component of the outer membrane that confers intrinsic resistance to many antimicrobial agents. LPS consists of the O-antigen, core polysaccharide, and a conserved lipid A moiety, which anchors the molecule to the membrane^2^. Polymyxins bind to lipid A by competitively displacing divalent cations such as Mg^2^⁺ and Ca^2^⁺, thereby disrupting membrane integrity in a detergent-like manner^2^. This membrane-targeting mode of action is mechanistically distinct from antibiotics acting on intracellular targets and may therefore impose different evolutionary constraints on resistance development.

Colistin was introduced into clinical use in the 1950s and approved by the U.S. Food and Drug Administration in 1959 for the treatment of Gram-negative infections^3^. Its application in veterinary medicine also spans several decades^4^. Despite earlier concerns related to nephrotoxicity and neurotoxicity and the availability of less toxic agents, colistin has re-emerged as a last-resort therapeutic option for severe multidrug-resistant infections^5,6^. Importantly, the historical toxicity concerns associated with colistin have not disappeared; rather, the drug has re-entered clinical use despite its narrow therapeutic window because therapeutic alternatives for multidrug-resistant Gram-negative infections are often limited.

For a long time, resistance to colistin was considered to arise predominantly through chromosomal mutations affecting lipid A biosynthesis or regulatory pathways^7^. This view was challenged by the discovery of plasmid-mediated mobile colistin resistance genes (*mcr*), which have since been detected in bacterial isolates from humans, animals, food, and environmental sources^8,9^. The global dissemination of *mcr* genes has raised serious concerns regarding the potential erosion of last-line therapeutic options^8^. In addition to mobile resistance determinants, chromosomal mechanisms involving the *pmrAB* two-component regulatory system represent a major pathway for colistin resistance development^10^.

Given the renewed importance of colistin in human medicine, its prudent use in veterinary settings is of critical importance. Strategies to reduce colistin exposure include strict biosecurity measures^11^ and evidence-based interventions that reduce the need for antimicrobial administration^12–17^. However, a key unresolved question remains whether prolonged colistin exposure promotes broader adaptive responses, such as cross-resistance to other antimicrobial classes, which could further exacerbate resistance risks.

From a veterinary microbiology perspective, commensal and opportunistic *E. coli* populations in food-producing animals represent an important reservoir for the emergence and dissemination of antimicrobial resistance, with potential consequences for therapy, biosecurity, and zoonotic transmission. In veterinary production systems, exposure to polymyxins may occur in spatially structured niches (e.g., intestinal gradients, biofilms), where selection can act locally and progressively. Therefore, a key practical question is whether stepwise colistin exposure alone is sufficient to promote broader collateral resistance patterns, or whether adaptation remains confined to polymyxin-specific pathways in the absence of horizontal gene transfer.

The present study aimed to investigate the phenotypic and genotypic consequences of stepwise colistin exposure in the model strain *E. coli* ATCC 25922. Using a spatially structured experimental evolution platform, we assessed whether increasing colistin concentrations drive compound-specific adaptation or induce broader resistance patterns, with particular emphasis on the emergence of cross-resistance and associated genomic changes.

## Results

### Phenotypic response to colistin selection across three independent runs

Across three independent MEGA-plate runs, all lineages reached the highest selection zone within 12 days. Colistin MIC increased from 0.5 μg/mL in the untreated isolate to 64 μg/mL in the highest selection zone, whereas MICs for the other tested agents remained unchanged across exposure levels (Supplementary Table S1). MIC profiles were identical across the three independent runs. For each exposure zone, one endpoint isolate per run was recovered (*n* = 3 isolates per zone). Because this shared phenotype was observed in all runs, one randomly selected representative isolate per exposure zone (0× , 1× , 10× , 100× , and 1000×) was carried forward for whole-genome sequencing. Accordingly, the genomic analyses below describe five endpoint representatives rather than all three parallel lineages recovered during the experiment.

Here and throughout the manuscript, 1× , 10× , 100× , and 1000× refer to nominal MEGA-plate selection zones scaled from the 0.125 μg/mL selection baseline, not to multiples of the parental MIC (0.5 μg/mL). No upward MIC shift was observed for any of the five β-lactams included in the panel.

### Baseline resistance-associated genomic repertoire of the sequenced representatives

Comparative screening against CARD strict hits identified a stable resistance-associated genomic repertoire across all five sequenced endpoint representatives. This repertoire was dominated by intrinsic/background loci and transport- or regulation-associated functions rather than by newly acquired resistance determinants.

Importantly, the presence of CARD-annotated resistance-associated loci was not interpreted here as equivalent to expressed phenotypic resistance across all annotated compound classes. In the ATCC 25922 background, many such matches represent intrinsic components, housekeeping-associated transport systems, or regulatory elements whose sequence presence alone does not establish functional activation or clinically meaningful resistance. Because the purpose of this analysis was to assess gene-content stability under colistin selection, the full gene-by-gene matrix is in Supplementary Table S2. Detailed supporting outputs underlying the gene annotations, full mutation calls, phage-proximity screening, and iMGE-proximity screening are provided in Supplementary Dataset S1.

Among the shared annotations, the β-lactamase-associated loci *ampC* and *ampH* were detected in all representatives, together with multiple efflux- and envelope-associated loci such as the *acrA–acrB–tolC*, *emrA–emrB*, and *mdtA–mdtB–mdtC* clusters. Importantly, both *ampH* and *emrB* were detected across all five sequenced endpoint representatives and were therefore not interpreted as exposure-specific gene acquisition events. These loci are listed in full in Supplementary Table S2 and are reported here only as a stable background repertoire rather than as evidence of broad phenotypic resistance.

### Genomic stability and mutation patterns under colistin pressure

Finalized assembly statistics for the five sequenced endpoint representatives are summarized in Table 1 using harmonized experimental and internal assembly identifiers. Average nucleotide identity (ANI) analysis comparing the untreated (0×) and highest-exposure (1000×) representatives yielded a value of 99.99%, supporting their close genomic relatedness despite phenotypic divergence in colistin MIC.

**Table 1:** Assembly summary for the five sequenced endpoint representatives of *Escherichia coli* ATCC 25,922.

Sample	Assembly code	Contigs ≥ 1 kb	Total length (bp)	Largest contig (bp)	GC (%)	N50 (bp)	N75 (bp)	L50	L75
0 × COL	coli_6	134	5,119,197	347,906	50.38	124,228	58,235	14	27
1 × COL	coli_7	142	5,119,626	348,005	50.38	120,956	57,987	15	29
10 × COL	coli_8	138	5,119,378	315,733	50.38	123,943	58,235	15	28
100 × COL	coli_9	148	5,118,276	338,069	50.38	132,233	57,487	14	29
1000 × COL	coli_10	178	5,115,822	248,991	50.4	97,369	41,384	18	39

Assembly statistics are reported directly for the five finalized endpoint assemblies used in the revised manuscript. Sample nomenclature is harmonized with the experimental exposure labels and the internal assembly identifiers listed in Supplementary Table S3. Zone labels are normalized to the 0.125 μg/mL selection baseline and do not represent direct multiples of the parental MIC.

COL, colistin; N50/N75, contig length such that 50%/75% of the assembly is contained in contigs ≥ that length; L50/L75, minimal number of contigs whose summed length reaches 50%/75% of the assembly.

For biological interpretation of adaptation under colistin selection, comparisons were centered on the sequenced 0× representative isolate as the experimental baseline. Relative to this untreated representative, exposed-zone genomes differed by only a small number of baseline-unique high-confidence annotated coding variants (Table 2). The 1× representative contained 10 such variants, the 10× representative 6, and the 100× and 1000× representatives 9 each. The number of candidate coding changes therefore remained limited across the sequenced endpoint genomes. Detailed supporting mutation outputs are provided in Supplementary Dataset S1.

**Table 2:** Baseline-unique high-confidence annotated coding variants in colistin-exposed representatives relative to the sequenced 0× representative isolate.

Exposure level	Complex	Deletion	Insertion	SNP	Total	Highlighted coding variant(s)
1×	4	0	1	5	10	*ompC* synonymous
10×	2	0	0	4	6	none highlighted
100×	4	0	0	5	9	*ftsI* missense
1000×	0	0	0	9	9	*ftsI* missense

Counts indicate variants absent from the untreated 0× representative and present in the indicated exposed representative. The table summarizes the number of baseline-unique complex variants, deletions, insertions, and single nucleotide polymorphisms (SNPs), together with highlighted coding variants discussed in the main text.

### Limited candidate coding changes in loci of possible relevance to susceptibility

Across the colistin-exposed isolates, only a limited number of candidate coding changes in loci of possible relevance to susceptibility were identified. Point mutations were identified in two genes, both involving loci commonly discussed in susceptibility changes via cell-envelope pathways (Table 3).

**Table 3:** Candidate coding variants in loci of possible relevance to susceptibility in *Escherichia coli* ATCC 25922 isolates recovered after exposure to increasing colistin selection zones.

Gene	Exposure level(s)	Nucleotide change	Protein change	Predicted effect	Product
*ftsI*	100× , 1000×	T-A	p.Asp566Glu	Missense variant	Penicillin-binding protein 3 (PBP3)
*ompC*	1×	G-A	p.Val166Val	Synonymous variant	Outer membrane porin OmpC

Only loci discussed in the main text are shown. Nucleotide and amino acid changes are reported relative to the reference genome.

In addition to the variants listed in Table 3, canonical colistin-resistance loci involved in lipid A modification and envelope regulation (*pmrA/pmrB, phoP/phoQ, mgrB, eptA/pmrC, arnBCADTEF,* and *lpxA/lpxC/lpxD*) were screened. No non-synonymous SNPs or indels were detected in these loci across the sequenced isolates, indicating that the observed phenotypic shift occurred without the typical genetic signatures reported for high-level polymyxin resistance.

A SNP in the *ftsI* locus was detected in the 100× and 1000× colistin-exposed representatives. Although *ftsI* is not considered a canonical determinant of colistin resistance in *E. coli*, envelope-associated changes involving this locus have been linked to altered susceptibility under antibiotic selection in other Enterobacterales^18^. In addition, a synonymous variant in the *ompC* porin gene was observed exclusively in the 1× colistin-treated sample. Because this was a synonymous change, it is reported descriptively only; porin-linked permeability changes are mentioned here solely as relevant biological context^19^.

## Discussion

The central result of this study is phenotypic rather than mechanistic: under the conditions tested, stepwise colistin selection increased colistin MIC without detectable collateral MIC shifts in the non-colistin panel. The genomic analyses are therefore interpreted as a constrained endpoint characterization of five representative isolates rather than as an exhaustive reconstruction of all adaptive routes available during the experiment.

Adaptation of *E. coli* ATCC 25922 to the highest colistin concentration (1000×) on the MEGA-plate required 12 days, consistent with a largely phenotypic response restricted to colistin under the conditions tested. In contrast to several other antimicrobial classes, colistin resistance is known to rely on a limited number of highly specific molecular mechanisms. Nevertheless, the rapid global dissemination of the plasmid-mediated *mcr-1* gene highlights the critical role of colistin use in veterinary medicine—particularly in intensive swine production—in accelerating resistance transmission between animal and human reservoirs^20^.

The mcr gene family encodes phosphoethanolamine transferases that modify the lipid A moiety of lipopolysaccharides by adding phosphoethanolamine, thereby increasing the net positive surface charge and reducing colistin binding affinity^21^. In the present study, no plasmid-mediated resistance determinants were detected, supporting that the observed phenotype evolved without acquisition of mobile colistin resistance genes.

Beyond plasmid-mediated mechanisms, colistin resistance can arise through chromosomal lipid A modification pathways, including *eptA*-mediated phosphoethanolamine decoration and related regulatory circuits^22^. Across the sequenced endpoint representatives, we identified only a limited number of coding changes with plausible relevance to envelope-associated adaptation. However, because one representative isolate per exposure zone was sequenced and no expression-level or functional validation was performed, the present dataset does not exclude broader regulatory responses, within-zone mutational heterogeneity, or adaptive processes not captured by endpoint short-read variant analysis. Accordingly, our genomic data support a limited coding signal in the sequenced representatives rather than a definitive absence of broader adaptive rewiring.

Efflux-mediated resistance to polymyxins has been described as species- and context-dependent, and multiple efflux systems have been implicated in antimicrobial peptide tolerance in Gram-negative bacteria^23^. In the present study, MIC testing showed no concurrent increase for the non-colistin agents included in the phenotypic panel. This pattern is not consistent with an obvious collateral MIC shift under the tested conditions; however, endpoint DNA sequencing alone is not sufficient to exclude efflux-related or other regulatory adaptation at the expression level.

A missense substitution in *ftsI* was detected in the 100× and 1000× representatives. Because *ftsI* encodes penicillin-binding protein 3, we specifically considered whether this finding might coincide with collateral β-lactam effects. However, the phenotypic panel already included five β-lactams, and none showed an upward MIC shift in the affected representatives. We therefore report the *ftsI* variant as a candidate envelope-associated change of uncertain relevance rather than as evidence of a measurable β-lactam collateral phenotype in this dataset^18^. A single synonymous *ompC* variant was detected exclusively in the 1× representative. Because this change does not alter the encoded amino-acid sequence and was not accompanied by any measurable MIC shift, it is reported descriptively but is not interpreted here as a confirmed driver of the phenotype^19^.

One of the most robust findings of this study was the absence of increased MIC values for the non-colistin agents included in the test panel, even in isolates recovered from the highest colistin selection zone. Within this controlled single-strain experimental system, the phenotypic response therefore remained restricted to colistin. Importantly, this conclusion should be interpreted within the boundaries of the present model, which did not include mixed populations, donor strains, or opportunities for horizontal gene transfer. Accordingly, the data support the absence of detectable collateral MIC shifts in the tested panel under the conditions used here, rather than a general exclusion of collateral adaptation in more complex ecological or clinical settings^24^.

Although this study used a reference strain, the findings inform risk assessment for colistin use in food animals where commensal *E. coli* populations experience localized exposure gradients (e.g., intestinal microenvironments and biofilms). The absence of collateral resistance under stepwise colistin pressure suggests that broad co-selection may depend more on the presence of transferable elements (e.g., *mcr*-bearing plasmids) than on colistin exposure alone.

Several features of the study design define the scope of inference. First, one endpoint isolate per run was recovered for each exposure zone (*n* = 3 isolates per zone), and phenotypic testing was performed across these independent lineages, whereas genomic analysis was based on one randomly selected representative isolate per zone. The genomic dataset should therefore be interpreted as representative rather than exhaustive. Second, because the study used endpoint short-read sequencing without transcriptomic or functional validation, the data do not exclude expression-level adaptation, structural variation, or other regulatory responses not captured by coding-sequence variant analysis. Third, the work was performed in a single reference-strain system without mixed populations or horizontal gene transfer, which makes it well suited for isolating chromosomal adaptation under controlled selection but limits extrapolation to more complex ecological settings. Variant discovery was performed with a single reference-based calling workflow; accordingly, low-frequency or caller-sensitive events should be interpreted cautiously.

## Materials and methods

### Bacterial strain

All experiments were performed using *Escherichia coli* ATCC 25922 (LGC Ltd., Teddington, UK), a widely used quality-control strain for antimicrobial susceptibility testing^25^. A complete ATCC 25922 genome assembly is publicly available and was used here as the species-matched external coordinate scaffold for reference-based comparisons. Given its well-characterized and stable baseline susceptibility profile, ATCC 25922 was selected as a standardized model to quantify de novo adaptation under controlled colistin selection. This strain was not used as a multidrug-resistant comparator; accordingly, the purpose of the baseline resistome analysis was to assess gene-content stability across exposure zones rather than to imply broad phenotypic resistance in the parental background.

### MEGA-plate preparation

Based on laboratory-specific requirements, a custom polycarbonate chamber (60 × 30 cm, 5 mm thickness; Innoterm Ltd., Budapest, Hungary) was constructed following the original MEGA-plate concept described by Baym et al.^26^ with minor practical adaptations^27^. Prior to each experiment, the chamber was disinfected using a 7.5% hydrogen peroxide solution prepared by diluting 30% hydrogen peroxide (VWR International Ltd., Debrecen, Hungary) with deionized water at a 1:3 ratio followed by chemical and UV decontamination under a laminar flow hood. The chamber was placed under a laminar flow hood, filled with the disinfectant solution, and the inner surface of the lid was wiped with a 0.9% sodium hypochlorite (NaOCl) solution (Merck KGaA, Darmstadt, Germany). After a 15-min incubation under sterile conditions, the disinfectant was removed using a vacuum system, followed by a 30-min exposure to ultraviolet light.

**Fig. 1:**
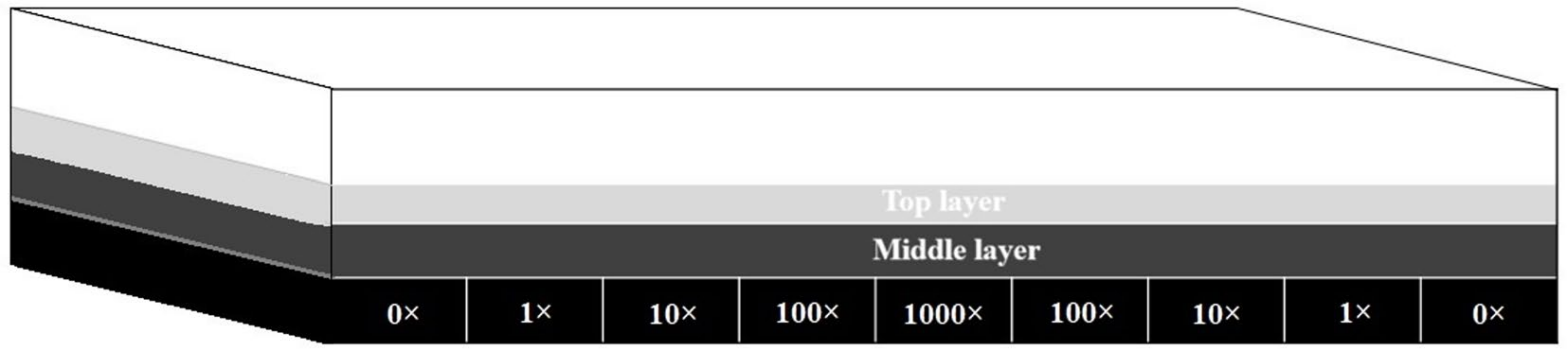
Schematic representation of the Microbial Evolution and Growth Arena (MEGA) plate system used in this study. The plate consists of three agar layers: a bottom layer containing nine physically separated compartments arranged symmetrically from the outer edges toward the center, a continuous solid agar middle layer limiting mixing between compartments, and a semi-solid top agar layer enabling bacterial diffusion and surface expansion. The five concentration classes used in the system were 0×, 1×, 10×, 100×, and 1000× , normalized to the 0.125 μg/mL MEGA-plate selection baseline; the mirrored layout therefore corresponded to 0× –1× –10× –100× –1000× –100× –10× –1× –0× . In this nomenclature, 1× , 10× , 100× , and 1000× denote nominal selection zones normalized to the 0.125 μg/mL selection baseline and do not correspond to direct multiples of the parental MIC.

The agar medium was prepared and poured in three distinct layers, each serving a specific function within the MEGA-plate system. The bottom layer consisted of nine physically separated compartments arranged symmetrically from the outer edges toward the center of the chamber. These compartments contained increasing concentrations of colistin (1× = 0.125 µg/mL; 10× = 1.25; 100× = 12.5; 1000× = 125 µg/mL). Owing to the mirrored layout of the plate, intermediate concentrations (1× , 10× , and 100×) were present on both sides of the central high-concentration zones, resulting in a total of nine compartments despite the use of five discrete antibiotic concentrations (Fig. 1).

The middle layer consisted of a continuous solid agar overlay that physically connected the underlying compartments while minimizing mixing of antibiotic concentrations between adjacent zones. The top layer was prepared as a semi-solid agar to facilitate bacterial diffusion and surface expansion along the established antibiotic gradient during incubation.

A 2% (w/v) BD Bacto Agar (VWR International Ltd., Hungary) was used for all layers except the top layer, which was prepared as a 0.28% (w/v) semi-solid formulation. LB-Lennox capsules (VWR International Ltd., Hungary) were added as a nutrient supplement at a ratio of one capsule per liter; based on a prior pilot experiment, an additional capsule was incorporated into the top layer to promote optimal surface growth. To prevent fungal contamination, cycloheximide (Merck KGaA, Darmstadt, Germany) was added at a final concentration of 64 µg/mL. For contrast enhancement, black acrylic paint (Artmie, Budapest, Hungary) was added at 4 mL/L to the media used for the lower and middle layers.

Finally, the *E. coli* strain was inoculated at both outer edges of the plate, and the assembled chamber was incubated at 37 °C until colony fronts reached the highest colistin zone.

### Antibiotic susceptibility testing

The MIC of the parental *E. coli* ATCC 25922 strain was determined in accordance with Clinical and Laboratory Standards Institute (CLSI) guidelines using the broth microdilution method^28^. Briefly, twofold serial dilutions of colistin were prepared in cation-adjusted Mueller–Hinton broth (CAMHB), inoculated with a standardized bacterial suspension (approximately 5× 10^5^ colony-forming units [CFU]/mL), and incubated at 37 °C for 18–24 h. The MIC was defined as the lowest antibiotic concentration preventing visible bacterial growth.

The baseline MIC of colistin for the parental strain was 0.5 µg/mL. To facilitate the gradual selection and spatial expansion of resistant subpopulations on the MEGA-plate, the initial selective concentration (1×) was set at one-quarter of the MIC (0.125 µg/mL). Subsequent antibiotic concentrations used in the MEGA-plate experiment (10× , 100× , and 1000×) were calculated relative to this 1× baseline.

Following a 12-day incubation on the MEGA-plate, during which bacterial growth progressed through zones containing up to the 1000× colistin concentration, samples were collected from each antibiotic exposure zone. To verify culture purity and exclude contamination, samples were streaked onto ChromoBio Coliform agar (Biolab Zrt., Budapest, Hungary). Colonies derived from a single CFU were subcultured on tryptic soy agar (Biolab Zrt., Budapest, Hungary) and preserved in Microbank cryovials (Pro-Lab Diagnostics, Richmond Hill, Canada) at − 80 °C until further analysis. The MEGA-plate experiment was performed in three independent parallel runs; for each exposure zone, one isolate per run was recovered at the endpoint and treated as an independent evolutionary lineage (*n* = 3 isolates per zone). For phenotypic testing, MIC values were determined for isolates from each run; because MICs were identical across the three runs, results are presented as a single common MIC profile. Prior to testing, frozen isolates were revived in 3 mL CAMHB and incubated at 37 °C for 18–24 h.

Antimicrobial susceptibility testing was performed using the broth microdilution method in sterile 96-well microtiter plates (VWR International Ltd., Debrecen, Hungary). Stock solutions (1024 µg/mL) of the tested antimicrobial agents—ceftriaxone, cefquinome, cefotaxime, ceftiofur, colistin, amoxicillin, neomycin, oxytetracycline, florfenicol, enrofloxacin and a potentiated sulfonamide combination—were prepared according to the manufacturer’s instructions and CLSI recommendations (Merck KGaA, Darmstadt, Germany). Twofold serial dilutions were prepared in CAMHB to achieve a final volume of 90 µL per well. The MIC panel included five β-lactams (amoxicillin, cefotaxime, ceftriaxone, ceftiofur, and cefquinome), allowing us to assess whether colistin selection was accompanied by collateral changes in β-lactam susceptibility.

Bacterial suspensions were adjusted to a turbidity equivalent to 0.5 McFarland using a nephelometer (Thermo Fisher Scientific, Budapest, Hungary), and 10 µL of each suspension was added to the wells to achieve a final inoculum of approximately 5× 10^5^ CFU/mL. MIC values were determined using a Sensititre SWIN automatic MIC reader and analyzed with Thermo Scientific Sensititre VIZION system software version 3.4 (Thermo Fisher Scientific, Waltham, MA, USA; https://www.thermofisher.com/uk/en/home/clinical/clinical-microbiology/antimicrobial-susceptibility-testing/sensititre-ast/vizion.html).

For whole-genome sequencing and downstream comparative genomics, one representative isolate per exposure zone (0× , 1× , 10× , 100× , and 1000×) was selected for sequencing; phenotypic MIC testing was performed on isolates from all three independent runs (n = 3 isolates per zone). The representative isolates were selected at random from the three independent runs because MIC profiles were identical across runs.

### Molecular genetic analysis

Genomic DNA was extracted from bacterial cultures using the Zymo Quick-DNA Fungal/Bacterial Miniprep Kit (Zymo Research, Irvine, CA, USA) according to the manufacturer’s instructions. Mechanical cell lysis was performed by bead beating using a TissueLyzer LT instrument (Qiagen GmbH, Hilden, Germany) at 50 Hz for 5 min to ensure efficient disruption of bacterial cells. Extracted genomic DNA was stored at − 20 °C until library preparation.

Whole-genome sequencing was performed using paired-end Illumina sequencing on a NextSeq 500 platform (Illumina, San Diego, CA, USA). Sequencing libraries were prepared using the Nextera XT DNA Library Preparation Kit, with indexing carried out using the Nextera XT Index Kit v2 Set A (Illumina, San Diego, CA, USA). Library preparation, amplification, purification, and quality control were conducted according to the manufacturer’s protocols. Libraries were quantified, pooled at equimolar concentrations, and sequenced following standard Illumina workflows.

### Bioinformatic analysis

To ensure one-to-one traceability between experimental labels, internal laboratory identifiers, de novo assembly names, and deposited genomic records, all sequenced endpoint representatives are cross-referenced in Supplementary Table S3. In this study, the sequenced representatives corresponded to the following internal assemblies: 0 × COL = coli_6, 1 × COL = coli_7, 10 × COL = coli_8, 100 × COL = coli_9, and 1000 × COL = coli_10. Supplementary Table S3 provides the experimental exposure label, internal sample code, internal assembly identifier, GenBank strain name, assembly filename, and the corresponding public record identifiers.

Read-level k-mer profiling was performed using GenomeScope 2.0 (https://genomescope.org/genomescope2.0/) to assess genome size, repetitiveness, and error structure^29^. Assembly completeness and taxonomic consistency were evaluated using CheckM v1.1.6 (https://github.com/Ecogenomics/CheckM) and Kraken 2 v2.1.3 (https://github.com/DerrickWood/kraken2)^30,31^. Average nucleotide identity between the untreated and highest-exposure representatives was assessed using FastANI v1.34 (https://github.com/ParBLiSS/FastANI)^32^.

Raw paired-end sequencing reads were subjected to quality control using FastQC v0.11.9 (https://www.bioinformatics.babraham.ac.uk/projects/fastqc/)^33^, fastp v0.23.2 (https://github.com/OpenGene/fastp)^34^, and BBDuk from BBTools v38.96 (https://jgi.doe.gov/data-and-tools/software-tools/bbtools/)^35^ to assess read quality, remove adapter sequences, and filter low-quality reads. Residual adapter and low-quality regions were trimmed using TrimGalore v0.6.7 (https://www.bioinformatics.babraham.ac.uk/projects/trim_galore/)^36^.

High-quality reads were assembled de novo into contigs using MEGAHIT v1.2.9 (https://github.com/voutcn/megahit) and SPAdes v4.0.0 (https://cab.spbu.ru/software/spades/)^37,38^, and assemblies were merged to improve draft genome contiguity using GAM-NGS v1.1b (https://github.com/vice87/gam-ngs)^39^. Assembly quality metrics were evaluated using QUAST v5.2 to confirm suitability for downstream analyses^40^.

Open reading frames were predicted from assembled contigs using Prodigal v2.6.3 (https://github.com/hyattpd/Prodigal)^41^. Acquired antimicrobial resistance genes were identified using RGI v5.1.0 (https://card.mcmaster.ca/analyze/rgi) and ABRicate v1.0.1 (https://github.com/tseemann/abricate) with reference to the Comprehensive Antibiotic Resistance Database (CARD)^42,43^. Only resistance genes meeting the CARD strict criteria and exhibiting ≥ 90% sequence identity and coverage were retained.

The genomic context of identified resistance genes was analyzed to assess potential association with mobile genetic elements using MobileElementFinder v1.0.3 (https://cge.food.dtu.dk/services/MobileElementFinder/). Plasmid origin of contigs was predicted using PlasFlow v1.1 (https://github.com/smaegol/PlasFlow), and potential bacteriophage-associated regions were identified using VirSorter v2.2.2 (https://github.com/jiarong/VirSorter2). Only mobile element-, plasmid-, or phage-associated regions exceeding 10 kb were considered for further evaluation^44–46^. Detailed supporting outputs for gene annotations, mutation calls, phage-associated proximity screening, and iMGE-associated proximity screening are compiled in Supplementary Dataset S1.

Chromosomal variants were investigated using a reference-based comparative framework. Because a separately sequenced pre-selection parental genome was not available, the sequenced 0× representative isolate served as the practical experimental baseline for adaptation-focused comparisons. The complete *Escherichia coli* ATCC 25922 assembly (GCF_000743255.1 / ASM74325v1) was used only as an external reference for coordinate-based annotation. Screening of chromosomal resistance-associated point mutations was performed using ResFinder 4.1 (https://cge.food.dtu.dk/services/ResFinder/), and genome-wide polymorphism tracking was carried out using Snippy v4.6.0 (https://github.com/tseemann/snippy). For biological interpretation, only annotated coding differences absent from the sequenced 0× representative and present in exposed representatives were considered. Accordingly, the variant counts reported in the manuscript do not represent the full set of raw differences detectable relative to the external reference genome, but the restricted subset retained for baseline-centered biological interpretation. To specifically assess canonical polymyxin-resistance pathways, variants were additionally screened in key loci involved in lipid A modification and envelope regulation, including *pmrA/pmrB, phoP/phoQ, mgrB, eptA/pmrC, arnBCADTEF,* and *lpxA/lpxC/lpxD*. Detailed supporting mutation outputs are provided in Supplementary Dataset S1.

## Conclusions

In this controlled single-strain MEGA-plate model, stepwise colistin selection increased colistin MIC from 0.5 to 64 μg/mL while MICs for the other tested agents remained unchanged. Among the five sequenced endpoint representatives, no plasmid-mediated colistin resistance determinants were detected, and only limited coding variation with plausible relevance to envelope-associated adaptation was observed. These findings support a compound-restricted phenotypic response under the conditions tested. However, because the genomic analysis was based on one endpoint representative per exposure zone and endpoint short-read sequencing without expression-level or functional validation, broader regulatory responses, efflux-associated adaptation, and within-zone genomic heterogeneity cannot be excluded. The principal value of the present study is therefore to define what can be concluded confidently from this controlled model: in the absence of horizontal gene transfer, stepwise colistin selection produced marked colistin MIC elevation without detectable collateral MIC shifts in the tested antibiotic panel.

## Supplementary Information

Below is the link to the electronic supplementary material.

Supplementary Material 1

## Data Availability

All publicly available sequence records generated in this study have been deposited under BioProject accession PRJNA1262446 (https://www.ncbi.nlm.nih.gov/bioproject/PRJNA1262446). These records include WGS master records, draft genome assemblies, and linked BioSample records for the five sequenced endpoint representatives. Supplementary Table S3 provides the exact correspondence between experimental exposure labels, internal sample identifiers, GenBank strain names, assembly filenames, and the associated public records. Detailed supporting outputs used for the genomic analyses are provided in Supplementary Dataset S1.
